# Assessing the individual roles of FII, FV, and FX activity in the thrombin generation process

**DOI:** 10.3389/fcvm.2022.1000812

**Published:** 2022-09-20

**Authors:** Cuicui Bai, Joke Konings, Marisa Ninivaggi, Marcus Lancé, Bas de Laat, Romy de Laat-Kremers

**Affiliations:** ^1^Department of Functional Coagulation, Synapse Research Institute, Maastricht, Netherlands; ^2^Department of Protein Engineering, Synapse Research Institute, Maastricht, Netherlands; ^3^Department of Platelet Pathophysiology, Synapse Research Institute, Maastricht, Netherlands; ^4^Department of Anesthesiology, Aga Khan University Hospital, Nairobi, Kenya; ^5^Department of Data Analysis and Artificial Intelligence, Synapse Research Institute, Maastricht, Netherlands

**Keywords:** thrombin generation, factor V, prothrombin, factor X, coagulation factors, calibrated automated thrombinography

## Abstract

Thrombin generation (TG) is known as a physiological approach to assess the hemostatic function. Although it correlates well with thrombosis and bleeding, in the current setup it is not sensitive to the effects of fluctuations in single coagulation factors. We optimized the calibrated automated thrombinography (CAT) method to quantify FII, FV and FX activity within the coagulation system. The CAT assay was fine-tuned for the assessment of FII, FV and FX by diluting the samples in FII-, FV-, or FX-deficient plasma, respectively, and measuring TG. Plasma FII levels correlated linearly with the ETP up to a plasma concentration of 100% FII. FV and FX levels correlated linearly with the peak height up to a plasma level of 2.5% FV and 10% FX, respectively. Sensitized CAT protocols were designed by adding a fixed volume of a pre-diluted patient sample to FII, FV, and FX deficient plasma in TG experiments. This approach makes the TG measurement dependent on the activity of the respective coagulation factor. The ETP or peak height were quantified as readouts for the coagulation factor activity. The intra- and inter-assay variation coefficients varied from 5.0 to 8.6%, and from 3.5 to 5.9%, respectively. Reference values were determined in 120 healthy subjects and the assays were clinically validated in 60 patients undergoing coronary artery bypass grafting (CABG). The sensitized CAT assays revealed that the contribution of FII, FV, and FX to the TG process was reduced after CABG surgery, leading to reduced prothrombin conversion and subsequently, lower TG.

## Introduction

The Calibrated Automated Thrombinography (CAT) is a global hemostasis assay that measures the thrombogenic potential of an individual (1). Since the introduction of the semi-automatic CAT method approximately 30 years ago (2), thrombin generation (TG) has gained popularity in assessment of coagulation (3–8). Over the last decade, many studies have shown the association of low TG and bleeding (9, 10), and high TG and thrombosis (11, 12). As a result of the clinical usefulness of TG in the prediction of bleeding and thrombosis risk (6), and the monitoring of patients receiving anticoagulation (13, 14) or transfusion products (15, 16), fully automated systems have been developed to measure TG in clinical laboratory settings (17, 18).

The TG test is a global coagulation assay, and therefore it is more sensitive to fluctuations in certain coagulation factors than others. Prothrombin and antithrombin, for example, are known to have a large effect on TG, whereas FV and FVIII only cause pronounced changes in the TG profile if their levels are far below the normal range (19). Moreover, the original CAT test results give the combined result of the interplay of all plasmatic components of the coagulation system, and subsequently changes in regular TG usually cannot be attributed to a specific coagulation factor (20, 21). Nevertheless, the function of individual coagulation factors is important, e.g., for monitoring treatment. Moreover, there is an increasing interest in the relationship between specific coagulation factor activity and thrombotic/bleeding diseases (22–25).

With modification, it has been shown that the CAT has the potential to measure the recombinant FVIII and FIX potency in hemophilia plasma (26–29). These findings demonstrate that there is potential to sensitize the CAT assay to individual coagulation factors. In this study, we set out to investigate the possibility to sensitize the CAT assay to the components of the prothrombinase complex (FII, FV, and FX).

## Materials and methods

### Patient and healthy subject samples

Healthy subjects (*n* = 120) were enrolled in the study after giving their full informed consent according to the Declaration of Helsinki and after approval of the local medical ethics board of Maastricht University Medical Center. Blood was collected on 3.2% sodium citrate (BD Vacutainer System). Platelet poor plasma was obtained by centrifugation twice at 2,630 g for 10 min and stored at −80°C until further use.

Additionally, 60 patients undergoing coronary artery bypass grafting (CABG) surgery were enrolled in the study. Blood was collected at two time points: before the start of the surgery and after the surgery and protamine administration. Blood was collected on 3.2% sodium citrate (BD Vacutainer System). Platelet poor plasma was obtained by centrifugation twice at 2,630 g for 10 min and stored at −80°C until further use.

### Thrombin generation

**Figure 1:**
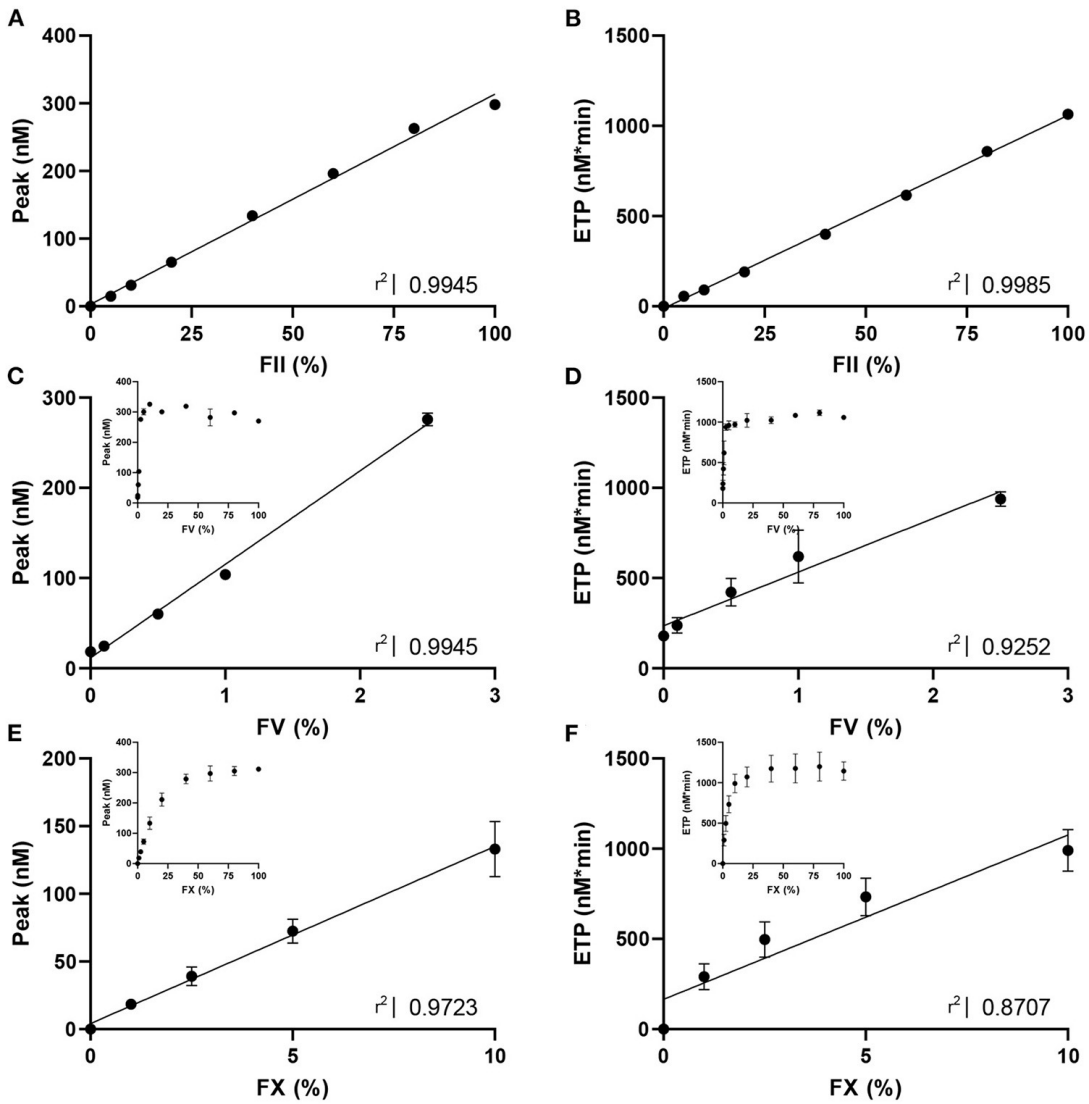
The dose response effect of FII, FV and FX on thrombin generation. TG triggered with PPP reagent high was measured in **(A,B)** FII-deficient plasma with varying concentrations of standard plasma (0, 5, 10, 20, 40, 60, 80, and 100% FII); **(C,D)** FV-deficient plasma with varying concentrations of standard plasma (0, 0.1, 0.5, 1, 2.5, 5, 10, 20, 40, 60, 80, and 100% FV); and **(E,F)** FX-deficient plasma with varying concentrations of standard plasma (0, 1, 2.5, 5, 10, 20, 40, 60, 80, and 100% FX). Data are shown as the mean ± standard deviation and linear regression R^2^ values are shown. For FV and FX, the linear part of the dose-response curve is analyzed with linear regression, and the whole range of ETP and peak are shown as insert in each panel. Statistical significance of the correlations was determined by Spearman correlation analysis, and all *p*-values were lower than 0.05.

TG without adaptations, as shown in Figure 1, was measured using the CAT method in a 96-well plate (Thrombinoscope B. V., Maastricht, Netherlands) (30). Each well contained 20 μL PPP Reagent High or 20 μL calibrator, 80 uL of platelet poor plasma sample, and 20 μL FluCa. Calibrator, PPP Reagent High and FluCa kits were purchased from Diagnostica Stago (France). Data were processed with dedicated software (Thrombinoscope B. V, Maastricht, Netherlands).

### Coagulation factor sensitized CAT assay

Coagulation factor sensitized CAT assays were performed as TG experiments, using a mix of prediluted plasma samples and commercial deficient plasmas. Pre-diluted plasma samples were prepared by diluting healthy individual or patient plasma samples in commercial plasma deficient in specific coagulation factors. The optimal conditions for plasma pre-dilution during the TG experiment and the ideal TG read-out parameter for the individual assays were determined separately for each assay, which resulted in three different measurement protocols for the coagulation factor sensitized CAT assays (Supplementary Figure 1). FII, FV, and FX deficient plasmas were purchased from Haematologic Technologies, Vermont, USA.

#### FII sensitized CAT assay

Each well of the 96-well plate contained 10 μL of undiluted patient sample, 70 μL of FII deficient plasma, 20 μL trigger mix and 20 μL FluCa. Calibration wells contained 10 μL of calibration plasma samples, which were prepared by mixing standard plasma to achieve calibration samples concentrations of 20, 40, 60, 80, 100, and 200% FII. TG was measured as described above, and a calibration curve was prepared based on the plasma FII activity and the corresponding ETP, allowing the calculation of the FII activity of patient plasma samples.

#### FV sensitized CAT assay

In the FV sensitized CAT assay, patient samples were diluted 1:20 in buffer. Each well contained 10 μL of diluted plasma sample, 80 μL of FV deficient plasma, 10 μL trigger mix and 20 μL FluCa. Calibration samples were prepared by mixing standard plasma with FV deficient plasma to achieve sample concentrations of 0.625, 1.25, 2.5, 5, and 10%. For patient samples, plasma was prediluted 1:20 to ensure that the FV levels within the calibrated range. TG was measured as described above, and a calibration curve was prepared based on the plasma FV activity and the corresponding peak height, allowing the calculation of the FV activity of patient plasma samples.

#### FX sensitized CAT assay

Each well of the 96-well plate contained 5 μL of patient sample, 75 μL of FX deficient plasma, 20 μL trigger mix and 20 μL FluCa. Calibration wells contained 5 μL of calibration plasma samples instead of patients samples, which were prepared by mixing standard plasma to achieve calibration samples concentrations containing 0, 25, 50, 100, and 150% FX. TG was measured as described above, and a calibration curve was prepared based on the plasma FX activity and the corresponding peak height, allowing the calculation of the FX activity of patient plasma samples.

### Statistical analysis

Statistical analysis was performed in Graphpad Prism 5.0 (GraphPad Software, Inc., California). Linear curve fit was used to establish the calibration curves. The sensitivity was defined as the lowest coagulation factor activity level generating a signal that could be statistically differentiated from the baseline activity in factor-deficient plasma. A *p*-value below 0.05 was considered statistically significant. Reference values were determined as the range between 2.5th and 97.5th percentile values determined in 120 healthy subjects. Differences between pre- and post-CABG samples were assessed using the Wilcoxon matched-pairs signed rank test.

## Results

To design CAT assays sensitized to the activity of FII, FV and FX, we first studied the dose-response effect of these coagulation factors on ‘regular' TG (Figure 1). TG was measured using PPP Reagent High in FII, FV or FX-deficient plasma spiked with increasing amounts of standard plasma, to achieve FII, FV or FX levels ranging from 0 to 100%, respectively. FII increased the ETP and peak dose-dependently for the whole range of concentrations (Figures 1A,B) and did not affect the lag time significantly. Complete deficiency in FII rendered an undetectable TG trace. In contrast, FV increased the peak height and ETP dose-dependently in the lower range (0–2.5% FV), but reached a plateau, respectively at 10% FV for the peak height and 20% FV for the ETP (Figures 1C,D). Additionally, the lag time decreased as FV increased. FX showed a similar effect on TG as FV, with a positive dose-dependent effect on ETP and peak height (Figures 1E,F). Alike FV, FX dose-dependently shortened the lag time.

**Figure 2:**
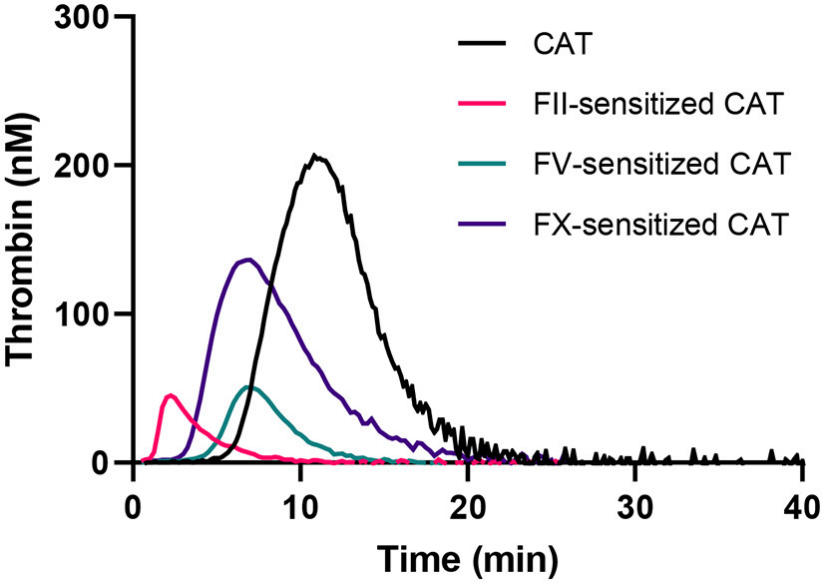
Thrombin generation curves generated in the standard CAT assay and the FII, FV, and FX sensitized CAT assays.

The obtained data was used to design CAT assays sensitized to either FII, FV or FX, by mixing (prediluted) patient plasma with FII, FV or FX deficient plasma, respectively. This approach enables us to make the TG measurement dependent on the patient-sample derived FII, FV or FX, and allows us to study the contribution of these specific factors to the thrombin generation measurement. Three separate protocols, as described in the methods section, were designed to ensure the most optimal determination of the contribution of FII, FV, and FX to TG. In the “regular” TG setup, the FII level showed the best linear correlation with the ETP, and FV and FX showed the best linear correlation with peak height in the low concentration range. Therefore, the ETP was used as the readout for the FII sensitized CAT assay, whereas the peak height was used for analysis of the FV and FX sensitized CAT assays. The correlation between FV and the peak height is only linear up to 2.5% FV. Subsequently, the patient plasma samples need to be prediluted for the FV-sensitized CAT assay, to achieve a final FV level below 2.5% during the TG measurement. Similarly, the plasma FX levels is linearly correlated with the peak height up to a level of 10%, and in the FX-sensitized CAT assay, the plasma is prediluted accordingly. The correlation of the ETP and FII was linear between 0 and 200% FII. Subsequently, patient samples do not need to be pre-diluted for the sensitized CAT assays. Figure 2 illustrates the appearance of the TG curves generated by the “regular” CAT method and the FII-, FV- and FX-sensitized CAT assays. The experimental variability of the coagulation factor sensitized CAT assays was determined by performing the assays in duplicate on 10 different days in a healthy donor sample (normal coagulation factor levels), and a sample from a patient using Vitamin K antagonists (low coagulation factor levels). A sample with low FV was prepared by diluting pooled normal plasma in FV deficient plasma, as Vitamin K antagonist therapy does not affect FV levels. For the FII sensitized CAT assay, the intra-assay coefficient of variation (CV_intra_) was 5.3 and 5.0%, respectively for the low and normal plasma sample. The inter-assay coefficient of variation (CV_inter_) were 4.8 and 5.9% for the low and normal sample, respectively. The CV_intra_ of the FV sensitized CAT assay were 8.7 and 8.6%, respectively for a low and normal plasma sample, and the CV_inter_ were 10 and 5.7%. The CV_intra_ of the FX sensitized CAT assay were 13.4 and 5.8%, in the low and the normal sample, respectively and the CV_inter_ were 16.0 and 3.5%. The lower limit of detection of the assays was 20, 0.25, and 10% respectively for the FII, FV and FX sensitized CAT assays.

### Assay validation in healthy subjects

**Figure 3:**
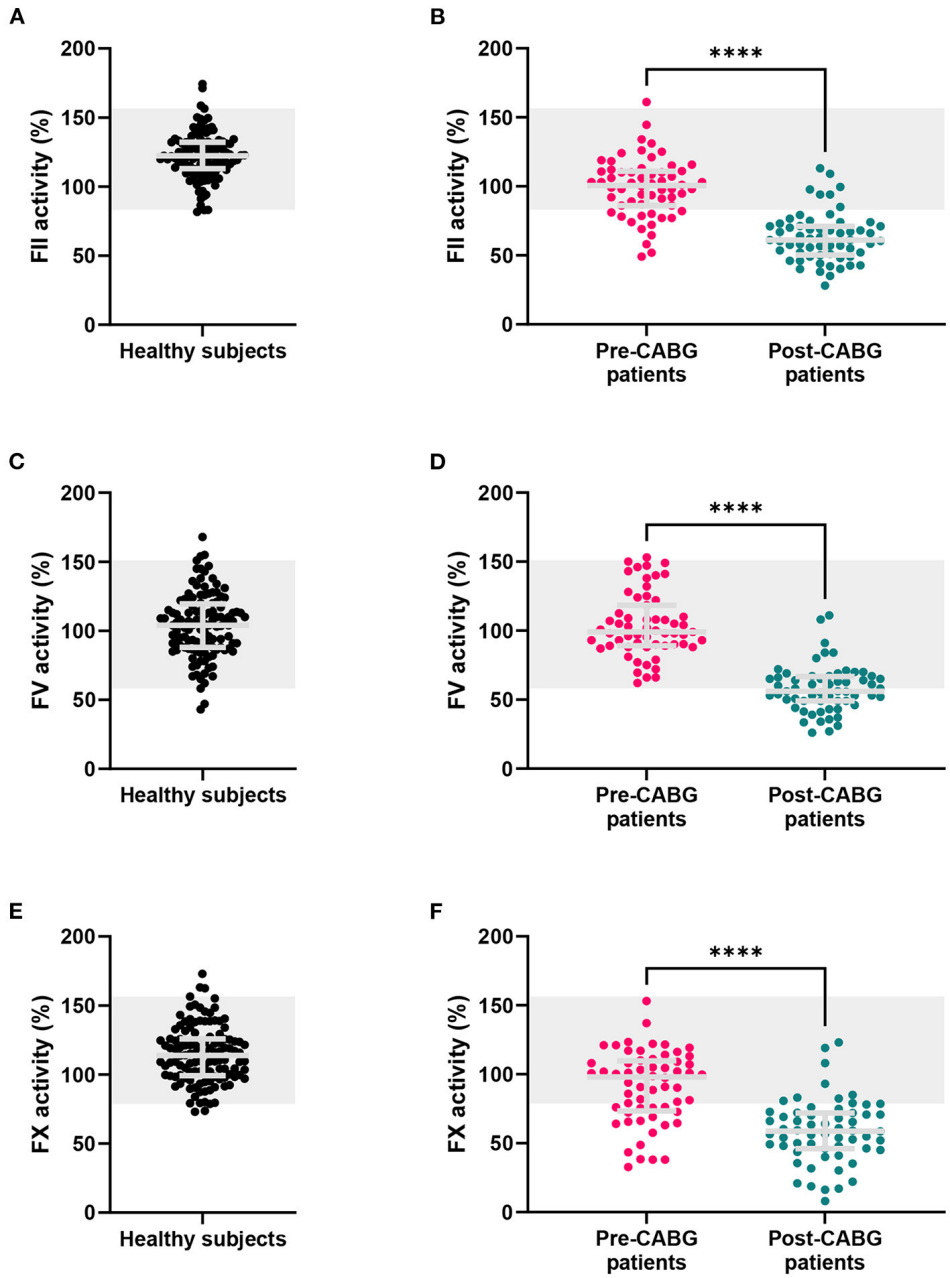
FII, FV and FX sensitized CAT assay results in healthy subjects, and patients before and after CABG surgery. **(A,B)** FII sensitized CAT assay results, **(C,D)** FV sensitized CAT results and **(E,F)** FX sensitized CAT were measured in healthy subjects and coronary artery bypass graft (CABG) patients before and after surgery. Results are depicted as individual data points with the median and inter-quartile range. The reference range for healthy individuals is marked in gray. ****p < 0.0001.

**Figure 4:**
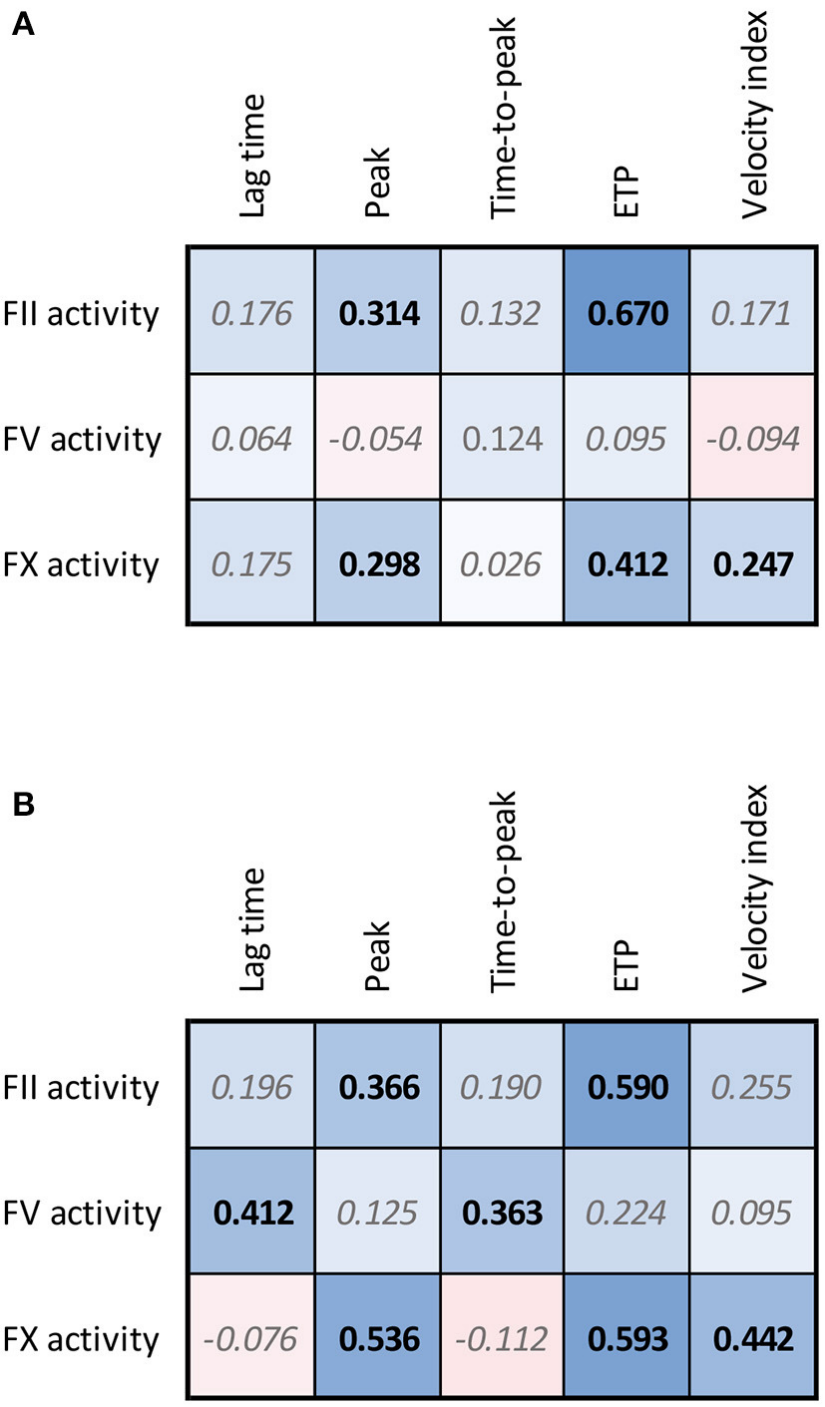
Correlation matrices for FII, FV, and FX sensitized CAT data and regular TG parameters in healthy subjects **(A)** and patients undergoing CABG surgery **(B)**. The spearman correlation coefficient is shown in the confusion matrix, and the color of the cells shows the strength of the association. Significant associations are shown in black font (*p* < 0.05).

The FII, FV and FX sensitized CAT assays were performed in 120 healthy subjects (Figure 3). In healthy subjects, the inter-individual CV was 13.5, 24.9, and 18.1% for the FII, FV and FX sensitized CAT assay, respectively. Reference ranges were determined for the three assays as the 2.5th−97.5th percentile values in 120 healthy individuals (Figure 3). FII has the narrowest range in healthy subjects, ranging from 85 to 157%. FV values showed the widest range, from 61 to 152% and FX ranged from 79 to 159%. Additionally, we investigated the correlation of the specific FII, FV, and FX activities in the CAT assay to the parameters of the original CAT assay in these 120 healthy subjects (Figure 4A). Both FII and FX activity are significantly and positively associated with the TG peak height and ETP in undiluted healthy subject samples. Furthermore, FX activity is positively associated with the velocity index. FV activity did not show a clear association with the original TG parameters in healthy subjects. Moreover, we determined the correlation of the TG parameters generated by the standard CAT assay and the FII-, FV-, and FX-sensitized CAT assays in healthy subjects (Supplementary Figure 2) and in post-CABG surgery patients (Supplementary Figure 3). The ETP measured in the standard CAT assay is the most important influencer of the ETP of the FII sensitized assay. For the FV- and FX-sensitized CAT assays, the association with the standard CAT assay are less pronounced, as the effect of FV and FX within the normal range are usually not detected by standard CAT. On the contrary in post-CABG surgery patient samples, in which the levels of FII, FV, and FX are reduced, the role of FV in the standard CAT assay becomes substantially larger, as indicated by the correlation of the peak height and ETP of the standard CAT TG and the FV-sensitized CAT TG.

### Clinical validation in patients undergoing CABG surgery

The clinical applicability of the sensitized CAT assays was investigated in patients undergoing a CABG procedure. CABG surgery is known to have a profound effect on the hemostatic balance, as the procedure causes blood loss, consumption of coagulation factors, heparinization and the administration of blood products to support coagulation. In this study, blood samples were taken before the start of the surgery and immediately after surgery and heparin neutralization.

The FII, FV, and FX sensitized CAT assays were performed (Figures 3B,D,F, respectively). The FII and FX activity was significantly lower in pre-CABG patients compared to healthy subjects (-19%, *p* < 0.0001 and −21%, *p* < 0.0001, respectively), and 23 and 32% of the pre-surgery patients were outside the normal range, respectively. The FV activity did not differ between healthy subjects and pre-CABG patients, and all patients were within the normal range prior to the start of surgery. After surgery, the FII, FV and FX activity was severely reduced compared to the pre-surgery coagulation factor activity. Moreover, the majority of patients had FII and FX activities below the normal range, post-surgery, and FV levels were outside the normal range in approximately half of the patients.

We further investigated the association of FII, FV and FX sensitized CAT assay results with the parameters of the original CAT assay performed on the pre-CABG surgery patient samples (Figure 4B). Similar to the healthy subjects, pre-CABG patients showed a significant positive association of the FII activity with the ETP and peak height quantified by regular CAT, and a positive association of FX with the ETP, peak height and velocity index. Additionally, a high FV activity was associated with a significantly longer lag time and time-to-peak.

**Figure 5:**
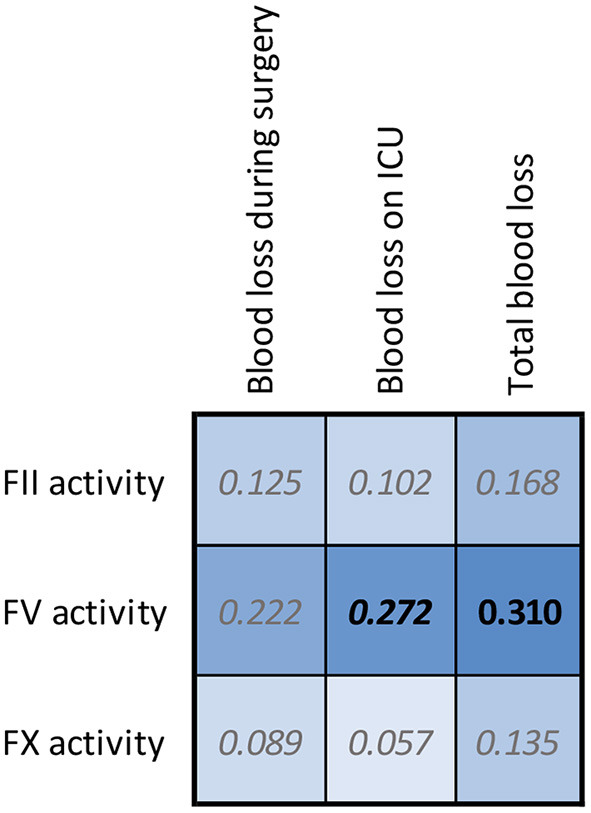
Correlation matrices for FII, FV, and FX sensitized CAT results in CABG patients and the blood loss during and after surgery. The spearman correlation coefficient is shown in the confusion matrix, and the color of the cells show the strength of the association. Significant associations are shown in black font (*p* < 0.05).

In Figure 5 we show the associations of the FII, FV and FX activity as measured by the sensitized CAT assays with peri-surgical blood loss. Pre-surgery FV activity measured by the FV sensitized CAT assay is significantly and positively associated with the post-surgery blood loss in the intensive care unit, and the total blood loss volume during and after the surgery. In contrast, FII and FX activity did not show a significant relationship with blood loss during or after surgery.

## Discussion

The TG assay is used in research labs worldwide to study the coagulation potential in research settings. Over the last decades, many reports have shown the association of lower TG and bleeding (3, 4, 8). Vice versa, higher TG has been associated with a higher risk of thrombotic events, such as venous thromboembolism, stroke, and myocardial infarction (5, 7, 31). As a results, efforts have been made to bring the TG assay into the clinical laboratories, to support clinical decision making (17, 32–34).

The TG assay was originally designed as a global test of hemostasis, including most of the relevant coagulation factors present in the blood. Additional methods have been developed for the TG assay, to determine the role of platelet count and function by measuring TG in platelet rich plasma (35–37), and to determine the added effect of other blood cells and components by measuring TG in whole blood (38–40). Moreover, thrombomodulin can be added to the TG assay to investigate the function of the anticoagulant activated protein C pathway (41–43), which causes the increased risk of thrombosis in women using oral contraceptives (21, 44). Furthermore, the sensitization of the TG assay for the detection of functional levels of FVIII and FIX, respectively in hemophilia A and hemophilia B patients, leads to the more accurate prediction of bleeding risk in these patients (27).

In the current study, we investigated the potential of sensitizing the TG assay to the factors of the prothrombinase complex, which is pivotal in the production of thrombin during TG. The formation of the prothrombinase complex is considered one of the pivotal steps in the hemostatic system. Homozygous deficiency of prothrombinase cofactor FV is rare and is associated with mild to severe hemorrhagic symptoms (45), whereas FX deficiency is one of the most severe rare coagulation defects, causing bleeding symptoms such as hematomas, and umbilical cord, gastrointestinal, and central nervous system bleeding (46). Moreover, prothrombin is essential to the formation of active thrombin, and the therapeutic lowering of its plasma level has been shown to be associated with bleeding episodes in multiple patient groups. We have previously shown that prothrombin and FX, and to a lesser extent FV, are important influencers of TG parameters (19). The current study shows that FII, FV, and FX correlate with the ETP and peak height, which is in line with previous findings that the ETP and peak height are most sensitive to variations of coagulation factor levels in plasma (47). Nevertheless, a reduction in the original TG peak height or ETP can be caused by a reduction of prothrombin, FV or FX, but also a reduction of other coagulation factors, such as FVIII or FIX (48), an increase of antithrombin levels (19), the use of anticoagulants (49) or other changes in the hemostatic system. In this study we set out to develop TG-based assays that can pinpoint changes in the function of FII, FV, and FX. Our results indicate that the developed TG assays have a high level of robustness to measure the activity of FII, FV, and FX. Furthermore, because this methodology is based on the original CAT assay, it is possible to use the assay in all labs that currently use the TG assay, in a similar fashion as the assessment of TM and APC sensitivity in the CAT assay. In addition, this assay can be performed alongside the routine CAT measurement, as the measurement conditions are the same, and only a small additional sample volume is required. Moreover, the development of these sensitized CAT assays can serve as an example to create other CAT assays sensitized to for example FVII, or anticoagulant factors such as antithrombin.

We further studied the clinical applicability of the newly developed assays in patients undergoing coronary artery bypass graft (CABG) surgery. Patients undergoing CABG surgery are known to be at risk for both bleeding and thrombosis due to an imbalance of coagulation factors caused by heparinization before the surgery, hemodilution during the surgery and protamine administration (50). We previously found that TG was lower in patients undergoing CABG surgery pre-surgery compared to healthy subjects (51), and the CABG surgery, as expected, lead to an even more pronounced difference in TG potential. Therefore, we quantified these changes further by applying the FII, FV, and FX sensitized TG assays.

Our results show that FII, FV, and FX are severely reduced after CABG surgery, reaching levels of 48%, 44%, and 49% of the original plasma levels, respectively. These findings are in agreement with the report of Coakley et al. describing similar decreases of FII, FV, and FX (52). Reports in an earlier cohort of patients undergoing surgery with cardiopulmonary bypass (CPB), revealed that the reduction of TG in CPB patients was mainly attributable to a reduction of prothrombin conversion (50). This indeed is in line with the current finding that all components of the prothrombinase complex FII, FV and FX are severely reduced in patients after CABG surgery. Moreover, we investigated whether pre-surgical FII, FV and FX activity in the sensitized CAT assay is associated with the amount of blood loss during the surgery and found that high FV activity is associated with significantly higher blood loss. Although this seems counterintuitive, the dual role of FV in the coagulation system has been described in the past (53). Correlations between changes in coagulation factor activity that are associated with an increased risk of bleeding are of clinical importance if pre-surgery coagulation factor activities are indicative of bleeding during surgery. Especially the combination of changes of several coagulation factor activity levels or hemostatic parameters resulting in an specific pro- or anticoagulant hemostatic profile could be of interest in the prediction of bleeding complications on an individual basis.

In conclusion, we have developed CAT-based assays that are specifically sensitized to the action of FII, FV and FX in the TG process. The assay was shown to be reproducible and experimental variation was low. Moreover, the assays were able to pinpoint the changes that occur during and after CABG surgery to the prothrombin conversion pathway, and more specifically to the reduced contribution of FII, FV and FX.

## Data availability statement

The original contributions presented in the study are included in the article/Supplementary material, further inquiries can be directed to the corresponding author.

## Ethics statement

The studies involving human participants were reviewed and approved by Medical Review Ethics Committee of Maastricht University Medical Center. The patients/participants provided their written informed consent to participate in this study.

## Author contributions

CB, RL-K, and BL conceived and designed the analysis. CB and JK collected the data. CB, ML, RL-K, and BL contributed data or analysis tools. CB, MN, and RL-K performed the analysis. CB, MN, RL-K, and BL prepared the first draft of the paper and the other authors revised the manuscript. All authors contributed to the article and approved the submitted version.

## Conflict of interest

CB, JK, MN, RL-K, and BL are employees of Synapse Research Institute. The remaining author declares that the research was conducted in the absence of any commercial or financial relationships that could be construed as a potential conflict of interest.

## Publisher's note

All claims expressed in this article are solely those of the authors and do not necessarily represent those of their affiliated organizations, or those of the publisher, the editors and the reviewers. Any product that may be evaluated in this article, or claim that may be made by its manufacturer, is not guaranteed or endorsed by the publisher.
