# Supplementary material for: Assessing the individual roles of FII, FV, and FX activity in the thrombin generation process
Source: Front Cardiovasc Med. 2022 Sep 20;9:1000812. doi: 10.3389/fcvm.2022.1000812 (PMC9530111; doi:10.3389/fcvm.2022.1000812)
Supplement: Supplementary file 1 [file Data_Sheet_1.PDF]

Supplementary Figures

Manuscript ID: 1000812

Research Topic: Advances in Thrombin Generation

Assessing the individual roles of FII, FV, and FX activity in the thrombin generation process

----

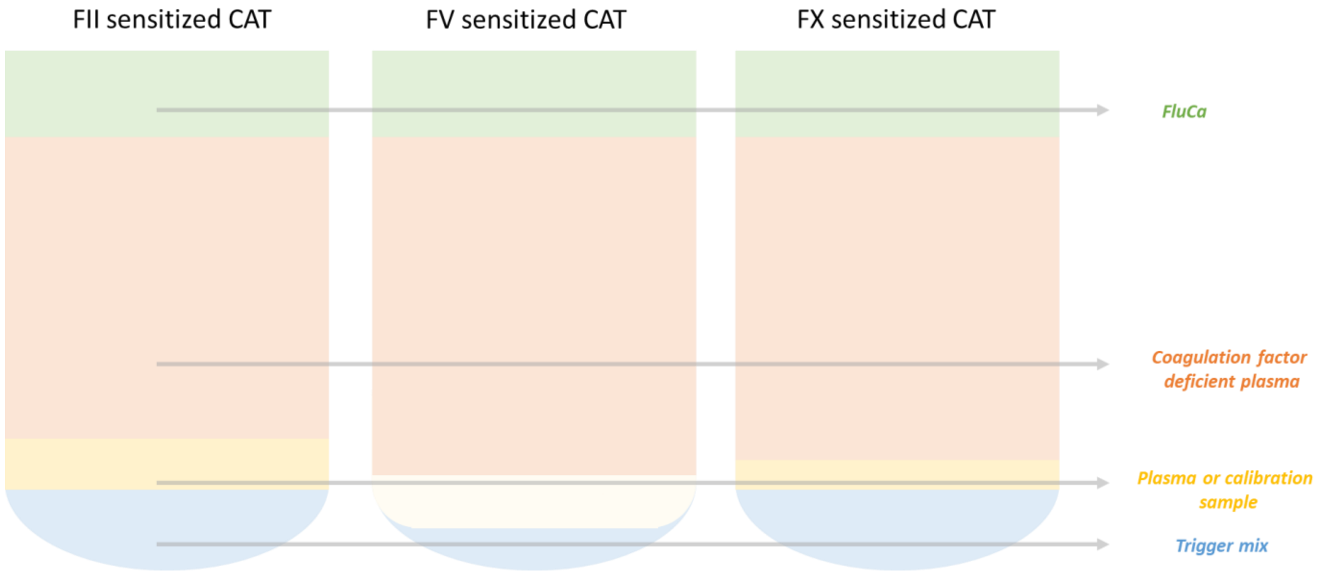

Supplementary figure 1: Schematic overview of the setup of the FII, FV and FX sensitized CAT assays.

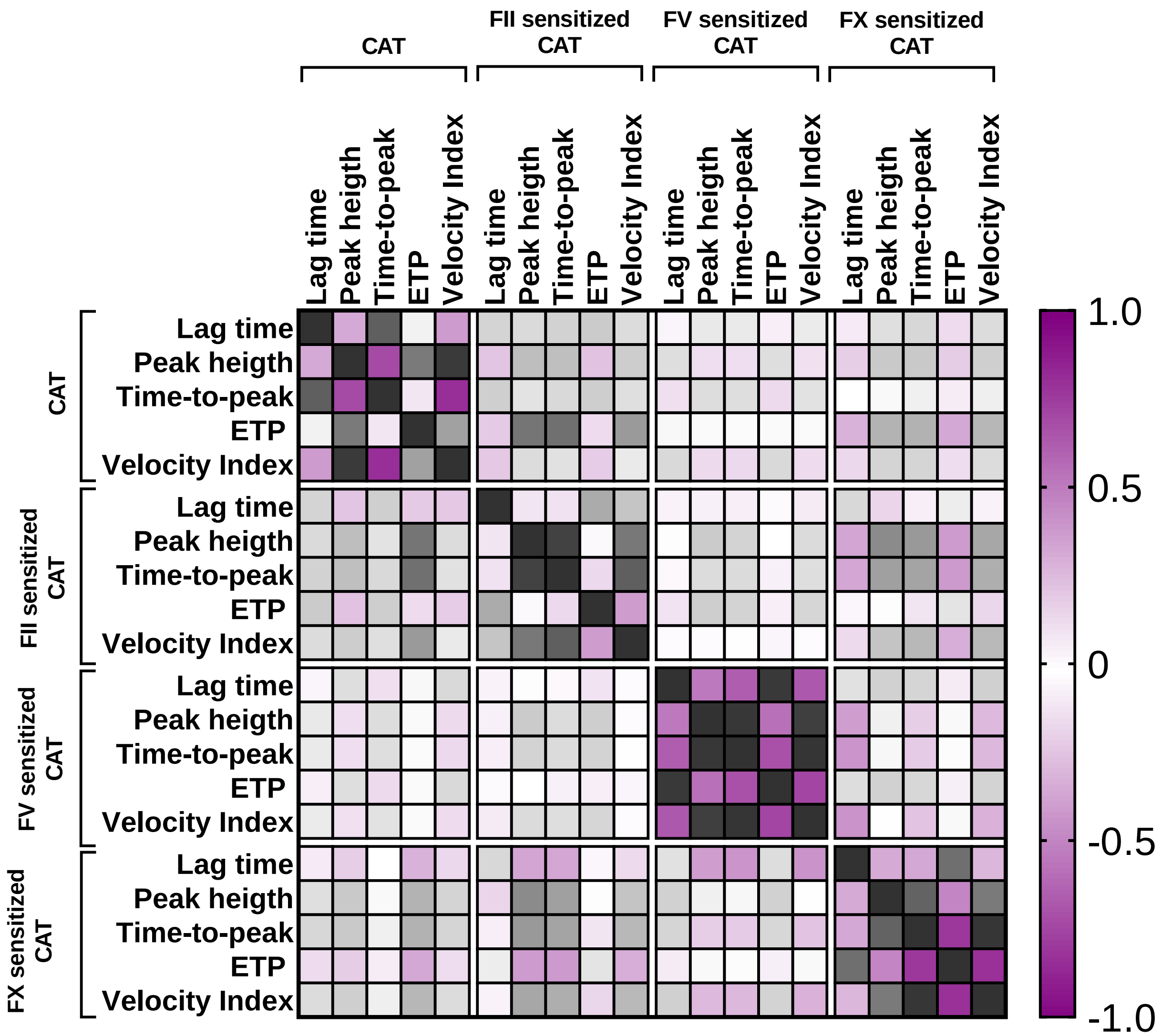

Supplementary figure 2: The correlation of the TG parameters generated by the standard CAT assay and the FII-, FV- and FX-sensitized CAT assay in post-CABG surgery patients.

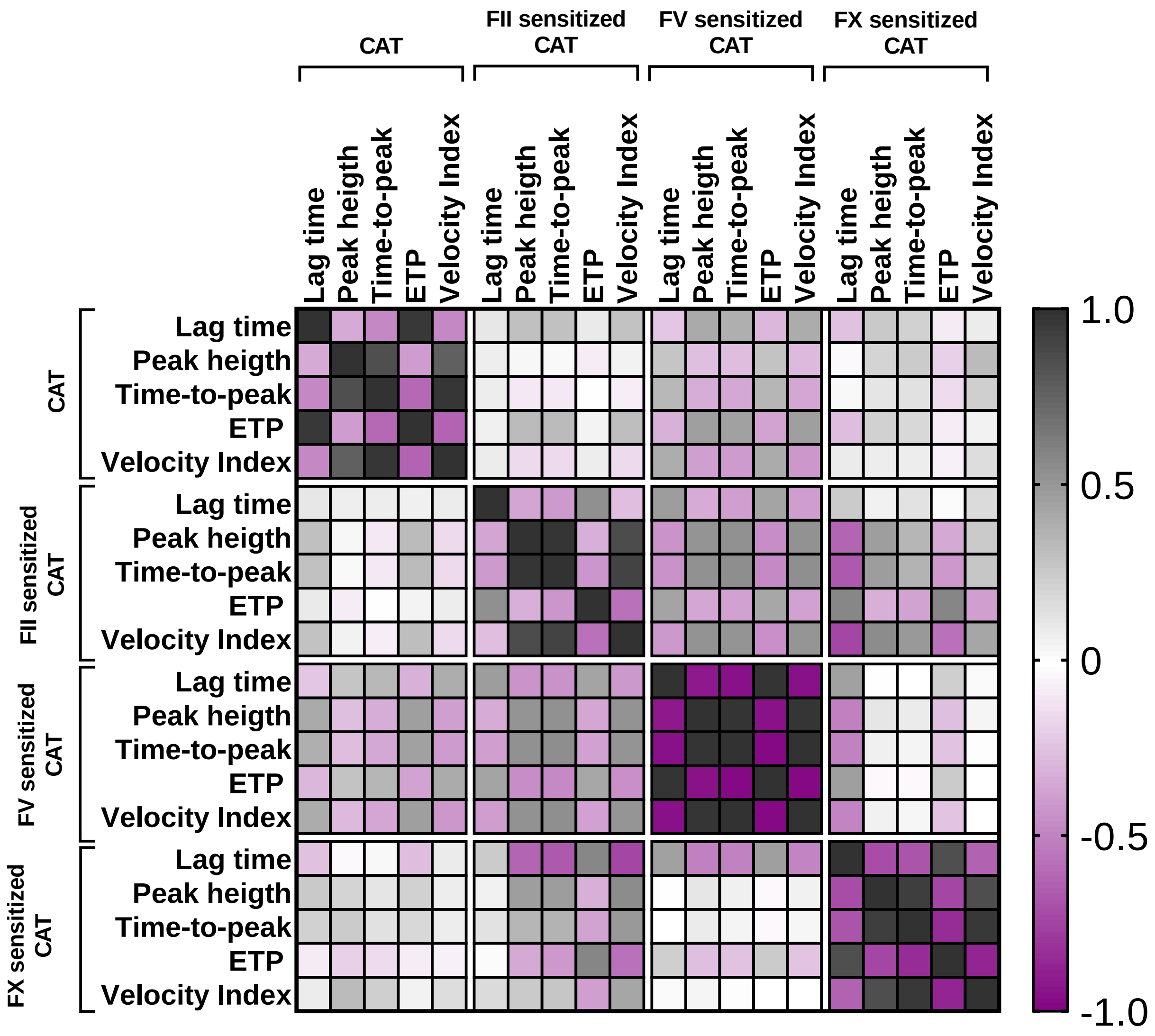

Supplementary figure 3: The correlation of the TG parameters generated by the standard CAT assay and the FII-, FV- and FX-sensitized CAT assay in post-CABG surgery patients.
